# A telemedicine instrument for remote evaluation of tremor: design and initial applications in fatigue and patients with Parkinson's Disease

**DOI:** 10.1186/1475-925X-10-14

**Published:** 2011-02-09

**Authors:** Mário C Barroso, Guilherme P Esteves, Thiago P Nunes, Lucia MG Silva, Alvaro CD Faria, Pedro L Melo

**Affiliations:** 1 Biomedical Instrumentation Laboratory State University of Rio de Janeiro, Rio de Janeiro, Brazil

## Abstract

**Introduction:**

A novel system that combines a compact mobile instrument and Internet communications is presented in this paper for remote evaluation of tremors. The system presents a high potential application in Parkinson's disease and connects to the Internet through a TCP/IP protocol. Tremor transduction is carried out by accelerometers, and the data processing, presentation and storage were obtained by a virtual instrument. The system supplies the peak frequency (fp), the amplitude (Afp) and power in this frequency (Pfp), the total power (Ptot), and the power in low (1-4 Hz) and high (4-7 Hz) frequencies (Plf and Phf, respectively).

**Methods:**

The ability of the proposed system to detect abnormal tremors was initially demonstrated by a fatigue study in normal subjects. In close agreement with physiological fundamentals, the presence of fatigue increased fp, Afp, Pfp and Pt (p < 0.05), while the removal of fatigue reduced all the mentioned parameters (p < 0.05). The system was also evaluated in a preliminary in vivo test in parkinsonian patients. Afp, Pfp, Ptot, Plf and Phf were the most accurate parameters in the detection of the adverse effects of this disease (Se = 100%, Sp = 100%), followed by fp (Se = 100%, Sp = 80%). Tests for Internet transmission that realistically simulated clinical conditions revealed adequate acquisition and analysis of tremor signals and also revealed that the user could adequately receive medical recommendations.

**Conclusions:**

The proposed system can be used in a wide spectrum of telemedicine scenarios, enabling the home evaluation of tremor occurrence under specific medical treatments and contributing to reduce the costs of the assistance offered to these patients.

## Introduction

Parkinson's disease (PD) is the most prevalent movement disorder, leading to symptoms including tremor, rigidity and bradykinesia [1]. According to the World Health Organization, in 2004 approximately 5.2 million people in the world presented this disease [2]. As a result of population growth and the increase in life expectancy, the number of people affected by PD will likely double in the next 25 years [3].

Rest tremor is the most common and easily recognized symptom of PD [4]. However, measuring the impact of therapeutic interventions on tremor features in PD poses a challenge because the severity of tremors may vary strongly during the day. Another important point is that most clinical rating scales are subjective. Additionally, the self-assessment of symptom severity by patients is not very reliable [5]. To overcome these shortcomings, objective assessment instruments have recently been developed. Es`kov *et al*. [6] described a research system for human micro movements that can be used to measure tremor. Hoff *et al*. [7] used a commercial portable multichannel recorder for quantitative continuous 24-hour monitoring of tremor while the patient is at home. Yang *et al*. [8] presented a portable device for the long-term measurement and recording of tremor in which these signals are stored in a compact flash memory card. More recently, Salarian *et al*. [9] developed an ambulatory system for quantification of tremor and bradykinesia in patients with PD that is based on miniature gyroscopes, while Engin [10] described an ambulatory instrument based on two axis acceleration sensors and Slack and Xianghong [11] presented a methodology for measuring the hand tremor of surgeons.

Due to the dramatic progress and concomitant decrease in the costs of electronics and telematics, many individuals have gained access to efficient communication solutions [12]. This allows for the possibility of growth in a specific telemedicine area, that of telemonitoring outside the hospital, i.e., at a patient's home or very close to it [12-14]. Portable electronic monitoring equipment has been shown to assist in the home monitoring of blood glucose [13], congestive heart failure [14], cardiovascular function [15] and geriatric patients who have a risk of falling [16].

Given the high relevance of telemonitoring PD patients, this problem has attracted a lot of attention recently, as can be observed in a recent conference [17-19] and currently ahead of print papers [20,21]. Patel *et al*. [22] used accelerometers to evaluate motor complications in persons with PD, and to predict the clinicians' estimates of disease symptom severity. Other signals have also been used in PD telemonitoring. Little *et al*. [23] assessed the practical value of discriminating healthy people from people with PD by detecting dysphonia. Tsanas *et al*. [24] proposed the use of speech tests in the evaluation of PD progression. Goetz *et al*. [25] tested the feasibility of a telemonitoring testing device in early-stage, unmedicated PD patients.

In this paper, we developed a wireless tremor monitoring system that integrates current personal digital assistant (PDA) and wireless communication technology, with a high potential to contribute in home monitoring of patients with Parkinson's disease.

A system for tremor recording and analysis is useful for at least two different user groups. Firstly, non-technical biomedical professionals who want to analyze tremor records in standardized daily clinical procedures, and secondly, users who work in different fields of tremor research. For the first group, the system should provide the possibility of displaying the results of a tremor analysis by means of a user-friendly front panel, whereas researchers usually need an open architecture with the flexibility to modify configurations and parameters.

## Methods

### System architecture

**Figure 1:**
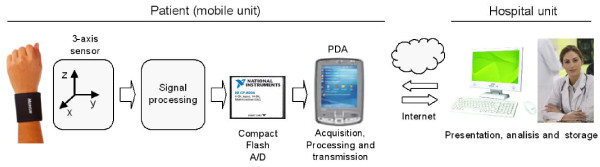
**Simplified block diagram of the telemedicine instrument for home monitoring of tremor**.

The general architecture of the instrument is reported in Figure 1. The telemedicine system consists mainly of two parts: 1) the mobile unit, which is set up around the patient to acquire the patient's tremor data and to receive medical recommendations, and 2) the hospital unit, which enables the medical staff to telemonitor the patient's condition and to send medical recommendations.

The management unit is either a fixed computer within an existing hospital network or a mobile laptop. The bidirectional transmission of data between the mobile unit and the hospital unit was implemented by the TCP/IP architecture. If the central station PC is in the same room as the mobile unit the communication can be accomplished simply by connecting a USB cable from the central station PC to the mobile unit (PDA).

### Hardware

**Figure 2:**
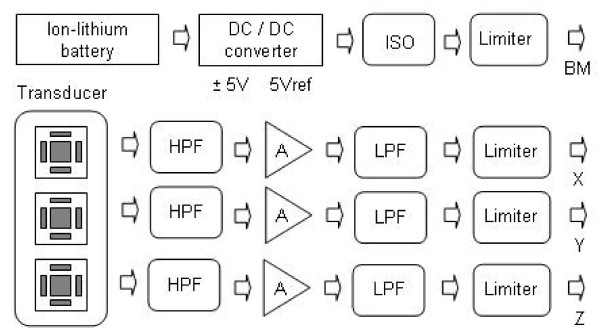
block diagram describing the transducer and signal processing stages HPF, high-pass filter, A, amplification, LPF, low-pass filter, ISO, isolator.

A detailed block diagram of the transducer and signal processing stages is shown in Figure 2.

The transducer used was a micromachined three axis accelerometer ADXL330 (Analog Devices), which is a small (4 mm × 4 mm × 1,45 mm) low power device that operates in a full-scale range of 3 g. Operating at 3.6 V, the sensitivity is 300 mV/g, with a nonlinearity of ±0.3% of full-scale and a bandwidth of 550 Hz.

The power source of the instrument was implemented using a rechargeable ion-lithium battery (KLIC-5001, Kodak Company) with a nominal tension of 3.6 V, current capacity of 1050 mAh and weight of 47 grams. A DC/DC converter (MAX680 - Maxim Integrated Products, Semiconducting Dallas) was used to get an adequate voltage supply to the circuits. The battery was initially adapted to the MAX680 to obtain symmetrical voltages of ± 10 V. The -10 V, in turn, is applied to a low power voltage regulator (MAX664) to be converted into -5 V, while the 10 V is converted to 5 V by a similar regulator (MAX666). The last IC cited includes a low battery detector that operates by comparing the battery voltage with an internal reference. The output of this comparator is an open drain field effect transistor, which can be damaged during the connection of the instrument to the external data acquisition system. We used an optocoupler in order to avoid this problem. A precision reference (REF 193, Analog Devices) was used to provide a stable 3.6-V reference to the acceleration transducers.

The human tremor vibrations are separated from continuous gravity acceleration by an analogue first-order high pass filter (0.3 Hz). Previous studies have shown that the tremor frequency in PD patients occurs below 30 Hz [7,26,27]. Thus, the X,Y,Z acceleration signals are amplified and adapted to low-pass filters (Butterworth, 4th order) with a cut-off frequency of 30 Hz used to eliminate external noises and the effect of aliasing. In order to increase the autonomy of the system, all of the analogue processing circuits were implemented using operational amplifiers with very low power consumption (OP 295, Analog Devices). With the aim of protecting the data acquisition board, the X,Y,Z acceleration signals as well as the battery-monitoring signal were maintained in a secure amplitude range by using limiter circuits constructed around zener diodes of 4.7 V.

These signals were adapted to a Compact Flash data acquisition card (NI CF-6005, National Instruments, Austin, Texas) with a resolution of 14 bits, four channels and a maximum sampling frequency of 200 kHz.

### Software

The system control programmes were developed in two environments: the PDA environment, based on the LabVIEW 8.2 PDA Module for Windows Mobile (National Instruments, Austin, TX) and the PC environment using LabVIEW 8.2 (National Instruments, Austin, TX). The graphical "G" programming used in LabVIEW™ allows a synthesis in icons of subroutines or complex algorithms, which can be interfaced by means of simple connections with other icons that compose a main programme. This feature makes the modification or inclusion of new subroutines easier, allowing the fast integration of new knowledge concerning the pathophysiologic mechanisms involved in Parkinson's disease without changing the structure of the main programme.

**Figure 3:**
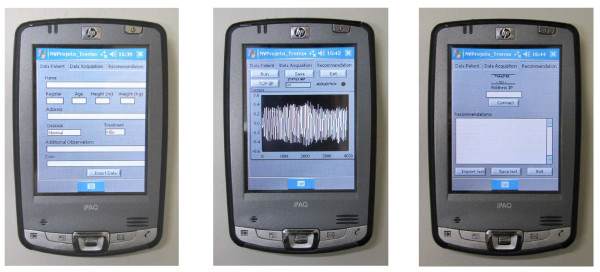
**Graphical user interfaces of the instrument in the PDAenvironment**. The software allows for the description of the characteristics of the patient and the results of the exam (A), and the data acquisition provides signal presentation and transference of the data between the patients'sPDA, the server and the remote computer (B). Medical recommendations may also be received by the patients (C).

In order to make clinical applications easier for non-technical personnel, dedicated user-friendly front panels were developed in the PDA environment. These interfaces are shown in Figure 3.

The software is composed of three main modules. The first includes subroutines to describe the characteristics of the patient and the results of the exam. The characteristics of the procedure may also be saved for further analysis, allowing easy evaluation of the evolution of medical treatments (Figure 3A). The second module performs acquisition of signals during the exam. The measurement of the three acceleration signals was performed simultaneously with a sampling frequency of 196 Hz. These signals are used to calculate the resultant acceleration vector, which is presented on the front panel of the virtual instrument. The acquisition occur over 20 s (Figure 3B). This module also includes routines that allow data transfer between the patients's PDA (the server) and the remote computer (the client). Also included in the software is a third module that allows the user to receive medical recommendations from the hospital unit (Figure 3C). This could provide significant benefits for the patient, allowing fast and easy adjustment of medical treatments.

The LabVIEW PDA environment presents all the necessary tools for elaboration of the programmes to be used with the patient's PDA (the server) and the remote computer (the client), in order to allow for data transfer using the Internet by means of a cellular connection. Communication between the PDA and the cellular (Nokia N95) was effected using the bluetooth protocol. Bluetooth is currently the most promising technology for wireless communication in an indoor environment. The advantage of Bluetooth compared to infrared technology is that the former does not require the sender to be in the line of sight of the receiver, as long as they are within the communication range, so the presence of an obstacle does not prevent communication, as opposed to infrared technology. Moreover, Bluetooth technology implements other features related to the security of communication, such as data encryption and user authentication, which can be particularly convenient when managing sensitive information such as clinical data. Another significant benefit for the patient is that medical recommendations obtained from the analysis of the results sent previously could also be sent. In the PDA environment, the hardware platform used was a Palmtop iPAQ HP hx2490 with 520 MHz, 64 Mb of RAM and 192 Mb of ROM, with the operating system Microsoft^® ^Windows Mobile^® ^5.0.

**Figure 4:**
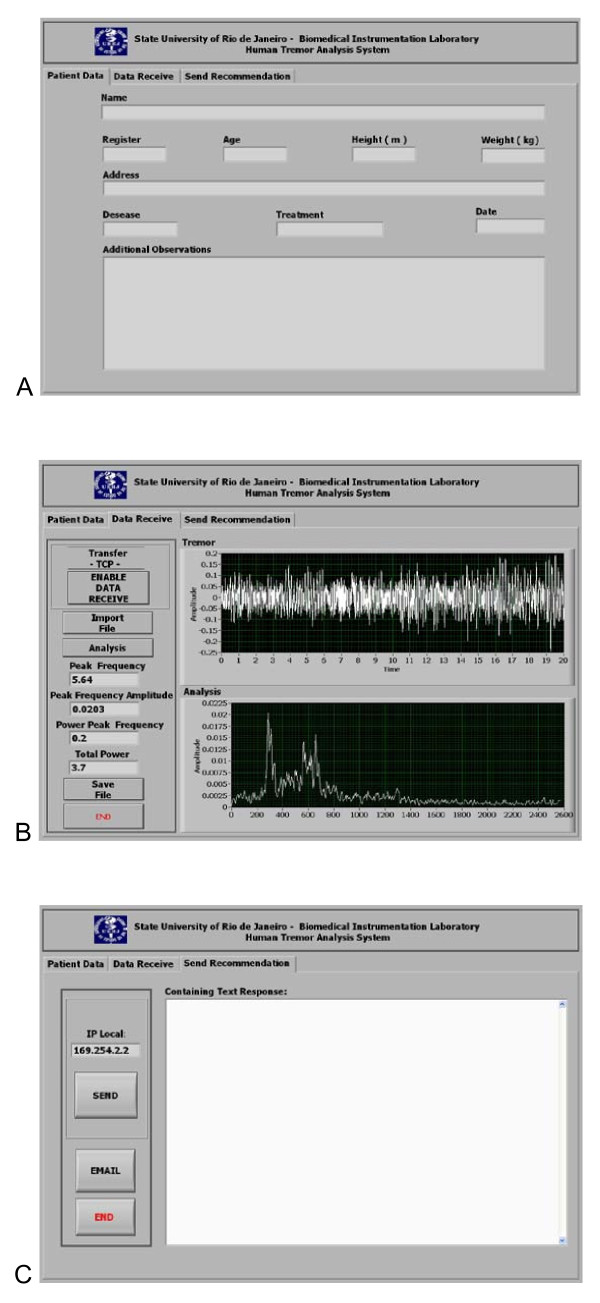
Graphical user interface of the instrument in the PC environment, which allows the user to observe the characteristics of the patient and the exam (A), a quantitative off-line analysis of the tremor signal (B), and a return of medical recommendations for the patient (C).

In the PC environment, on the other hand, the hardware platform was constituted by an Intel Core 2 Duo 2.2 GHz computer with 4 GB of RAM, a hard disk of 80 GB and Microsoft Windows XP operating system. A user-friendly front panel was also developed to be used in the PC environment. This interface is shown in Figure 4. Initially, the characteristics of the patient, of the exam and of the medical procedure are presented to the user (Figure 4A). The second module provides a graphical description of the tremor signal against time (Figure 4B), allowing the user to detect artifactual events such as speaking and inappropriate corporal movements by visual analysis.

The user may also perform a quantitative off-line analysis of the measured accelerations. To this end, initially the average value of the vector sum is removed. Then, the amplitude spectrum was estimated using the available fast Fourier transform (FFT) routines in LabVIEW 8.2. The FFTs were computed using blocks of equal length (10 s, 1960 points), which resulted in a spectral resolution of 0.1 Hz [10]. A fifty percent overlap was used in order to increase the number of independent data blocks and reduce random errors. Data blocks were multiplied by a Hanning window and then processed by Fourier analysis. The values from each 10-second period (three blocks) were averaged, and the result of the test was calculated as the mean of these measurements. These points describe acceleration as a function of frequency and are expressed in units of g (Figure 4). Based on the mean spectrum, the program uses an algorithm available in LabVIEW 8.2 that automatically searches for the frequency at which the signal of highest intensity occurs (peak) and estimates the peak frequency (fp) and the power around it (Pfp). The power is estimated considering three frequency lines before the peak frequency line, the peak frequency line itself, and three frequency lines after the peak (eq. 1).

(1) $$\text{Pfp=}\sum_{\text{fp-0}\text{.3Hz}}^{\text{fp+0}\text{.3Hz}}\text{P(f)}$$

Peak amplitude (Ap) was also obtained using a built-in LabVIEW algorithm. As the oscillations in pathological tremors are only approximately sinusoidal [28], the programme also includes the estimate of the total power (Ptot). This parameter was obtained by considering the power throughout the frequency range studied (eq. 2).

(2) $$\text{Ptot=}\sum_{\text{0}\text{.3 Hz}}^{\text{30 Hz}}\text{P(f)}$$

After the patient evaluation, based on the previously described parameters, the physician may also send medical recommendations back to the patient's PDA using the panel described in Figure 3C. This facility contributes to a fast and easy adjustment of medical treatments.

### Instrument access, data security, error handling protocols

For security reasons, algorithms for proper authorization use of the virtual instrument were developed. For both the fixed and portable subsystems, all users are required to enter a user name (login) and a password to access the system.

Great care was taken to ensure secure data transmission capability over public data networks. Firstly, we chose Bluetooth technology to perform the data transmission between the PDA and the telephone because Bluetooth protocols performs frequency hopping over 79 channels, each one displaced by 1 MHz (in the Industrial Scientific and Medical band, between 2.402-2.480 GHz), at 1600 hops per second. Bluetooth protocols also implement reliable and robust data encryption and user authentication solutions. More specifically, we used Bluetooth version 1.2, class 2 (range of 10 m) with transmission speed of 1 Mbit/s. Bluetooth security procedures are based on a L2CAP (Logical Link Control and Adaptation Protocol) implementing translation of data into secret code based on the SAFER (Secure And Fast Encryption Routine) and a block cipher encryption algorithms. Therefore, eavesdroppers cannot read the contents during data transmission. Data security and integrity are also guaranteed by pairing devices, creating a shared secret known as a link key. This link key is stored by both devices, and then the identity of the devices is cryptographically authenticated previously to starting communicate.

Data interchange in the Internet was performed using International Mobile Telecommunications-2000 (IMT-2000) standards, also known as 3G. We chose to use this technology because of the enhanced cryptography methods used to implement confidentiality and integrity algorithms. In addition to the data security cryptography used in Bluetooth and IMT-200 standards, a custom encryption algorithm was also implemented in our laboratory using the LabVIEW 8.2 environment. This algorithm allows both mobile unit and hospital unit to perform additional encryption.

All built-in error handling routines available in LabVIEW 8.2 PDA Module were used to ensure that data were completely transferred and without transmission artifacts. During data transmission, these routines could detect file errors before sending it. In these cases, an error message is displayed to the user in order to correct the error or to repeat the tremor measurement. If an error occurs during TCT-IP data exchange, it is canceled and an error message describing the error displayed to the user, allowing the starting of a new data exchange procedure.

When data are received, error handling protocols first check the number of bytes to read. If the number of bytes is fewer than the requested number that arrives after the maximum time expected to perform communication (25 s), a timeout error is reported and data may not be used. The software automatically send a message to the patient requesting the re-sending of the file and ensuring data integrity.

### Laboratory *In vivo *tests in normal subjects during fatigue

The ability of the proposed system to detect human tremor was initially validated in a simulated study in normal subjects. Thus, we performed a comparative analysis on the results obtained in 10 normal subjects (28.6 ± 9.1 years, 72.5 ± 9.2 kg, 172.6 ± 7.6 cm, 6 male) in three conditions: (1) with the arm extended without load; (2) in a similar way supporting a mass around 1.6 kg and (3) with the arm extended after the release of the load. These manoeuvres are known to provoke tremor associated with muscular fatigue [29,30]. In these evaluations, the transducers had been adapted to the right wrist of the volunteers. The measures were made with the volunteers standing with their right arm extended.

### Laboratory *In vivo *tests in patients with Parkinson's disease

**Figure 5:**
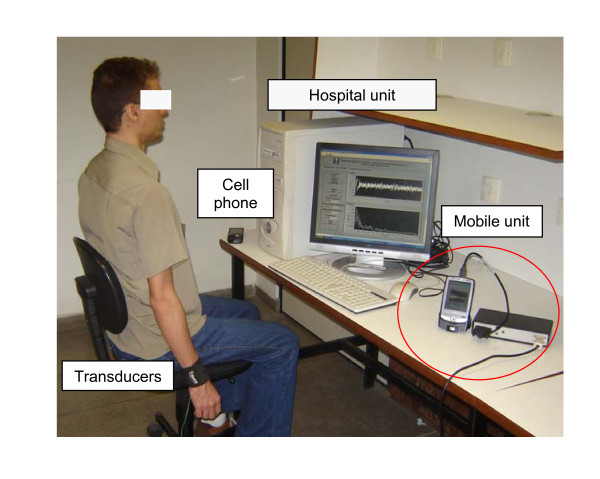
**Picture of the telemedicine instrument**. The figure shows the typical positioning of the volunteer during the exam, the PDA, the hospital unit, the mobile unit and the transducer positioning on the patient's wrist.

The system was also validated by a comparative analysis between 10 normal subjects (28.5 ± 8.5 years, 66.4 ± 12.7 kg, 170.4 ± 9.0 cm, 5 male) and 10 subjects with Parkinson's disease (69.8 ± 7.1 years, 69.4 ± 12.0 kg, 156.0 ± 8.9 cm, 3 male). In this comparison, the transducers had been attached to an elastic strap and adapted to the wrist associated with the arm that had first presented tremors due to Parkinson. The measures were made with the individuals comfortably seated in a chair, with feet flat on the ground. The measured arm was extended along the body, with the hand of the segment not assessed resting on the respective leg (Figure 5). The exams began after a period of approximately one minute of adaptation to the apparatus, and volunteers were asked to not wear bracelets, watches or rings during the measurements.

In vivo experiments were performed according to protocols approved by the Ethics research Committee of the State University of Rio de Janeiro. Informed consent was obtained from all volunteers before inclusion in the study. The data are expressed as means ± SD.

As can be seen in Table 1 in the Results section, tremor in PD patients usually occurs at a frequency between 4 and 6 Hz, while in normal subjects fp is around 2 Hz. In order to contribute to the diagnosis of tremor, this study also included the evaluation of the powers in the cited frequency ranges (1-4 Hz, Plf, and 4-7 Hz; Phf). The ability of the studied parameters to identify abnormal movements in Parkinson's patients was evaluated by means of receiver operating characteristic (ROC) analyses, which were conducted using MedCalc 8.2.

**Table 1:** Tremor parameters in normal subjects and in patients with Parkinson's disease.

	fp (Hz)	Ap (g)	Ppf (mV^2^)	Ptot (mV^2^)	Plf (mV^2^)	Phf (mV^2^)
Controls (n = 10)	2.6 ± 1.5	0.0021 ± 0.0011	0.003 ± 0.003	0.022 ± 0.0068	0.007 ± 0.003	0.006 ± 0.004
Parkinson (n = 10)	5.4 ± 1.5	0.091 ± 0.0791	6.7 ± 9.5	42.1 ± 48.1	9.2 ± 14.0	24.6 ± 25.8
p	< 0.01	< 0.01	< 0.001	< 0.001	<0.001	<0.001
Sensitivity (%)	100	100	100	100	100	100
Specificity (%)	80	100	100	100	100	100
AUC	0.895	1	1	1	1	1

Values are presented as means ± SD. SD = standard deviation; fp = peak frequency; Ap = peak amplitude; Pfp power around the peak frequency; Ptot = total power; Plf = power in low frequency; Phf = power in high frequency; AUC = area under the receiver operating characteristic curve.

Data from, Afp followed a normal distribution and therefore the standard t-test was used. A non-parametric test (Mann-Whitney U Test) was applied for non-normal distributions (fp, Pfp, Ptot, Plf, Phf). These analyses were performed using Origin 6.0 software. A p value of less than 0.05 was considered as statistically significant.

### Internet transmission tests

The first tests for Internet transmission were conducted in the laboratory by sending information from the mobile unit to a bench top computer. Five tests were performed, and the simulated results and recommendations were exchanged via the Internet.

For an initial proof of concept on remote monitoring and medical information exchange, typical measurements were performed, realistically simulating clinical conditions. To this end, tremor measurements were performed in normal subjects during fatigue. These subjects were located in three private homes in Rio de Janeiro, Brazil, and this information was sent to our laboratory in Rio de Janeiro. Simulated medical recommendations were also sent back to these subjects.

We assessed the overall network speed between the PDA and the PC. The test was conducted transmitting typical files over the Internet (tremor exams: approximately 150 kB; recommendations: approximately 1.0 kB). The transmission speed was evaluated once an hour, for a period between 9:00 and 18:00.

## Results

A prototype of the overall system has been designed and implemented. The photograph in Figure 5 depicts the developed system.

The mobile unit, which includes the tremor signal acquisition module and a wireless PDA, is compact and lightweight (600 g). The current consumption of the module is 2.5 mA. Considering this consumption, the battery used (1050 mAh) and a mean measurement time of 5 minutes, the signal acquisition module has autonomy of approximately 420 measurements. The Pocket PC, on the other hand, can run under its own power for at least 4.3 hours. Considering the same mean measurement time, the PDA has autonomy of 51 measurements.

### Laboratory *In vivo *tests in normal subjects during fatigue

**Figure 6:**
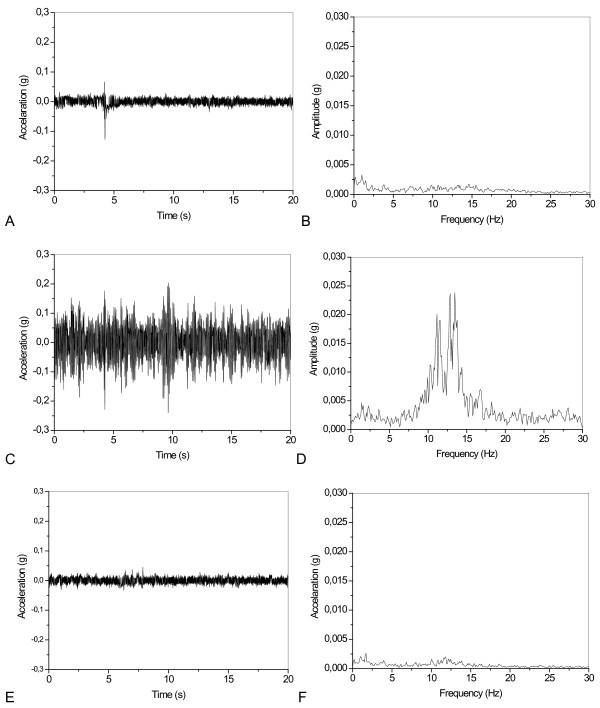
**Representative examples of the typical results obtained in normal subjects before (A,B), during (C,D) and after fatigue (E,F)**.

**Figure 7:**
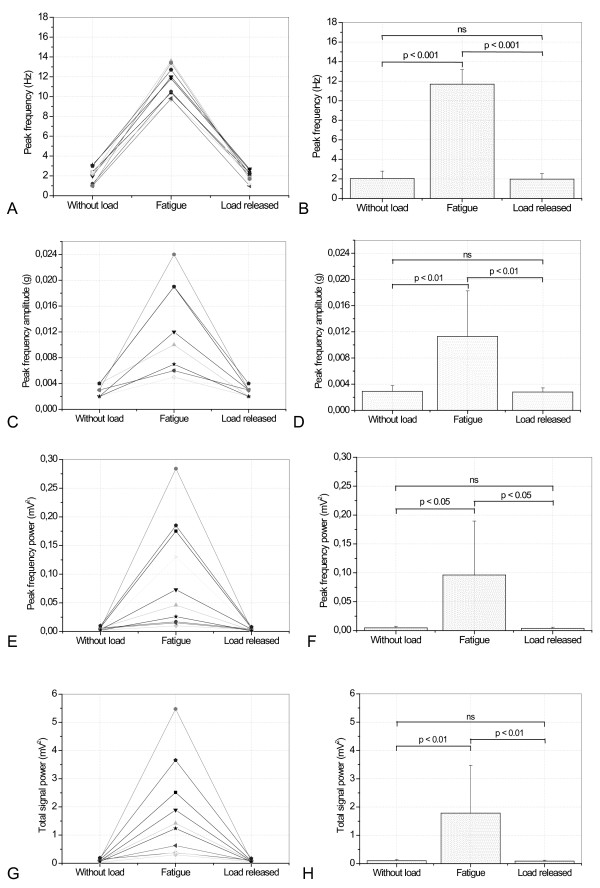
Individual values (A,C,E,G) and mean values (B,D,F,H) of the tremor parameters before fatigue, during fatigue events, and values obtained after fatigue (E).

A representative example of the typical results obtained in these studies is presented in Figure 6. Figure 7A,C,E and 6G present results comparing basal tremor parameter values before fatigue, during fatigue events, and after fatigue in each of the studied subjects. Mean values of fp and Ap (Figure 7B and 7D) presented highly significant increases during fatigue (p < 0.001 and p < 0.01, respectively). Mean values of Pfp (Figure 7F) and Ptot (Figure 7H) also presented significant increases during fatigue (p < 0.05 and p < 0.01, respectively).

The removal of the mass and the consequent fatigue remission caused a significant decrease in fp and Ap (p < 0.001 and p < 0.01, respectively), which became similar to the values observed before fatigue (Figure 7B and 7D; p = ns). On average, Pfp (Figure 7F) and Ptot (Figure 7H) also presented significant decreases after removal of the mass (p < 0.05 and p < 0.01, respectively), returning to values similar to those detected before fatigue (p = ns).

### Laboratory *In vivo *tests in patients with Parkinson's disease

**Figure 8:**
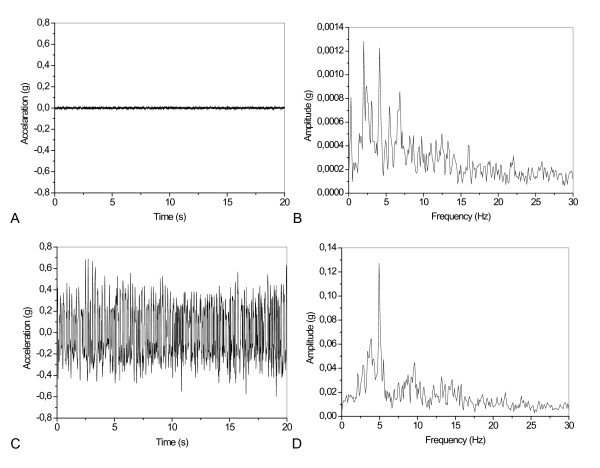
**Examples of results obtained with the proposed system for the evaluation of the human tremor**. Time and amplitude spectrum of a normal individual (A,B) and similar signals from a patient with Parkinson's disease (C,D). Note the difference in the amplitude spectrum scales.

Figure 8 shows typical results describing the influence of Parkinson's disease on the tremor signal in the time (A) and frequency (B) domains.

Table 1 describes the influence of Parkinson's disease on the studied tremor parameters. The mean values of fp and Ap presented highly significant increases in Parkinson's disease patients (p < 0.01 and p < 0.01, respectively). The Ppf, Ptot, Plf and Phf values were higher in Parkinsonian patients and presented the most significant differences among all parameters studied (p < 0.001).

The amplitude in the peak frequency, Ppf, Ptot, Plf and Phf achieved perfect accuracy (AUC = 1) in the detection of tremors in patients with Parkinson's disease, with a sensitivity of 100% and a specificity of 100%.

### Internet transmission tests

In the five initial Internet communication tests conducted in the laboratory, there were no errors in the comparison of the files saved on the PDA and those sent via the Internet. In the second tests, involving three simulated exams in normal subjects located in three private homes in Rio de Janeiro, we were able to receive tremor waveforms on the client computer, perform the spectral analysis on the tremor signals and obtain tremor parameters without errors. On the other hand, the remote PDA user could also adequately receive simulated medical recommendations.

The network speed varied with time (mean ± SD = 3.6 ± 1.6 kBps; maximum = 6.4 kBps; minimum = 0.9 kBps). Therefore, the PC receives tremor data sent from the PDA in a mean time of 41.6 s and a maximum time of 2.8 min. Recommendation files, on the other hand, may be sent from the PC and received in the PDA in a mean time of 0.3 s and a maximum time of 1.1 s.

## Discussion

It is expected that telemedicine, and especially home telecare, will gain increasing diffusion in the coming years [31]. In this context, Lin *et al*. [32] recently developed a wireless PDA-based system to telemonitor a patient's vital signs continuously during intrahospital transport.

We developed a medical instrument for remote evaluation of tremor, with high potential use in home monitoring of patients with Parkinson's disease. Indeed, the treatment of several disorders can benefit from home tremor monitoring solutions, such as multiple sclerosis, stroke, traumatic brain injury and a number of neurodegenerative diseases that damage or destroy parts of the brainstem or the cerebellum. Measurements of tremor at home without hospitalisation could reduce costs, enable follow-up of tremor results after specific medical treatments and allow for observation of the effects of environmental and behavioural factors such as physical activity, smoking and diet on tremor results.

While home monitoring enables longitudinal studies, it also places stringent constraints on the system: cost, size, unobtrusiveness, ease of use, and maintenance of signal quality. The mobile system developed in the present work is simple to use and does not hinder the patients. In fact, the mobile unit is lightweight and easy to carry. This subsystem may even be worn attached to a belt around the waist. The power autonomy allows more than 50 exams. During tremor measurements, the patient briefly interrupts their daily activities, sits on a chair and the data acquisition unit and the PDA are placed on a table. The system has a user-friendly interface that records patient information, collects and displays tremor biosignals and supports wireless communication. The instrument does not present hardware-based controls, which greatly simplifies its use by nontechnical personnel. On the other hand, the PC unit has an easy-to-use interface that is used to input patient information, display tremor biosignals and to launch an analysis panel. When needed, the FFT analysis can be performed on the tremor signal by clicking a button. In addition, several parameters can be calculated to describe the tremor signal characteristics.

### Laboratory *In vivo *tests in normal subjects during fatigue

Initially, the proposed system was validated in a simulated study under controlled conditions in normal subjects evaluating its ability to detect tremor under conditions of fatigue. In the absence of weight (Figures 5 and 6), a tremor of low amplitude, power, and frequency around 2.0 Hz was observed. In agreement with the physiology involved [29], the studied parameters fp, Afp, Pfp and Pt increased in all of the studied volunteers during the fatigue process (Figure 7). This resulted in significant increases in the mean values of the cited parameters. Subsequent removal of the weight resulted in the reduction of all the studied parameters. It is interesting to note that after removal of the weight and the end of fatigue events, it is possible to observe a significant reduction of the tremor parameters and a return to the initial condition before fatigue.

These results are consistent with physiology fundamentals [29] as well as with previous results obtained with strain gauge measurements [30] and clearly describe the increased tremor frequency and amplitude associated with the process of muscular fatigue. This provides substantial evidence that the proposed system can be useful in the study of physiological tremor in normal individuals.

### Laboratory *In vivo *tests in patients with Parkinson's disease

In the second experimental validation study, the results obtained in 10 parkinsonian subjects were compared with 10 control subjects. In line with previous results [10,28], we found higher tremor frequencies, amplitudes and powers in patients with Parkinson's disease (Figure 8, Table 1). These results are also consistent with the findings of Hashemi *et al. *[33] and a recent clinical review that pointed out that parkinsonian tremor occurs at a frequency between 4 and 6 Hz [4].

Using the studied parameters, it was possible to clearly differentiate the abnormal tremor events in Parkinson's patients. The performance of the studied indices was evaluated by means of ROC curves [34,35]. Analysis of ROC curves is performed by plotting sensitivity versus 1-specificity for each possible cut-off level. This way, the larger the area under the curve (AUC), the more valid the diagnostic test. According to the literature, ROC curves with AUCs between 0.50 and 0.70 indicate low diagnostic accuracy, AUCs between 0.70 and 0.90 indicate moderate accuracy, and AUCs between 0.90 and 1.00 indicate high accuracy. An AUC > 0.80 is usually considered adequate for clinical use [34,36]. Thus, fp reached acceptable values for clinical use, identifying patients with abnormal tremor with a sensitivity of 100% and a specificity of 80% (Table 1). Higher accuracies were obtained when the Afp, Ppf, Ptot, Plf and Phf were considered, which allowed a perfect identification of tremor in normal subjects and in patients with Parkinson's disease (AUC = 1). Thus, these parameters may be useful in the clinical detection of abnormal tremor in parkinsonian patients. These results also confirmed that this system could be a convenient alternative to ambulatory exams.

### Quality control of home measurements of tremor

Tremor recording by the patient at home may present artifacts associated with acceleration signals due to speaking or other movements of the patient during tremor exams. In our system these events can be easily detected by the patient by visual analysis of the tremor signal, which is displayed on the screen after the measurement. Even if the patient sent measurements with errors, these errors may be easily detected in hospital unit since the developed software allows biomedical professionals to obtain a detailed visual description of possible artefactual events in the time and frequency domains. In the presence of these events, patients can be promptly advised and asked to perform and send a new tremor exam. The possible transmission of duplicate files by the patient is another reason to discard tremor measurements in the central hospital unit. To prevent this error, a routine in LabVIEW 8.2 was developed in order to compare the received file with others previously sent by the same patient. In the event of sending a duplicate file, the patient is automatically notified by an automatic message explaining what happened and asking permission to send the file.

### System architecture and transmission tests

An important aspect of the system, as it is designed to be used by non-technical biomedical professionals, is that users generally expressed positive comments about ease of use of the instrument and in configuring the proper settings. Laboratory communication sessions proved to be robust, and were performed correctly in all of the tests.

Communications between the mobile unit and the central unit were designed to be bidirectional. All laboratory and remote tests were conducted using a commercial phone network. Tests realistically simulating clinical use showed that it was possible to send tremor data from the mobile unit and also to receive simulated medical recommendations without any errors. Although the network speed varied with time (mean ± SD = 3.6 ± 1.6 kBps), resulting in a maximum transmission time of 2.8 min, in practice, the observed velocities may be considered adequate for the desired application.

### Limitations of the study

There are three potential limitations in the present study. The first one is seen in Table 1, where the standard deviations of Ppf, Ptot are almost as large as the mean values. This may induce the reader to think that these parameters are difficult to be trusted. Since the ability of the cited parameters to detect the differences between normal and patients was demonstrated by the high values of sensibility, specificity and AUC (table 1), these high standard deviations are not a problem in the present study.

The second potential limitation concerns the clinical validation of the proposed system in home monitoring of abnormal tremor in PD patients. The present work is dedicated to describe a new instrument and to a feasibility study showing that the instrument is useful in tremor measurements, and can be used in remote evaluations. It is not a demonstration that it will be clinically successful in monitoring Parkinson's patients. This would require a different kind of study, which is clearly beyond the scope of the present paper.

Finally, the data sample of only 10 patients with PD is limited. A larger sample of subjects would be necessary to draw more robust conclusions.

## Conclusion

A novel system for tremor evaluation, with potential use in home monitoring of subjects with Parkinson's disease, was developed in the current study. Using this instrument, the acquisition and analysis of tremor signals can be performed remotely through the Internet. On the other hand, the user could also receive medical recommendations. The system is small enough to be easily transported.

The ability of the system to detect abnormal tremor was initially validated by a fatigue study in normal subjects and then in a preliminary *in vivo *test in parkinsonian patients. The proposed system may be useful in a wide spectrum of telemedicine scenarios, possibly enabling follow-up of tremor under specific medical treatments and reducing the cost of assistance offered to these patients.

Based on these promising results, future work includes a clinical trial in which we will perform a follow up in well-defined groups of Parkinson's disease patients in order to evaluate the clinical contribution of the home monitoring approach in improving the patient's outcomes.

## Competing interests

The authors declare that they have no competing interests.

## Authors' contributions

MCBJ, GPE and TPN developed the instrumentation, analyzed the data, and drafted the manuscript. LMGS and ACDF provided data, subject identification and participated in the data analysis process. PLM conceived the study, mentored all participants and helped to draft the manuscript. All authors have read and approved this manuscript.
